# Biomimetic Anti-Adhesive Surface Microstructures on Electrosurgical Blade Fabricated by Long-Pulse Laser Inspired by Pangolin Scales

**DOI:** 10.3390/mi10120816

**Published:** 2019-11-26

**Authors:** Chen Li, Yong Yang, Lijun Yang, Zhen Shi

**Affiliations:** 1 College of Mechanical and Electrical Engineering, Shaanxi University of Science and Technology, Xi’an 710021, China; yanglijun@sust.edu.cn (L.Y.); 20150312105@sust.edu.cn (Z.S.); 2 State Key Laboratory of Transient Optics and Photonics, Xi’an Institute of Optics and Precision Mechanics, CAS, Xi’an 710119, China; yangyong@opt.ac.cn

**Keywords:** biomimetic scales, laser microfabrication, anti-adhesion, anti-friction, electrosurgical blade, 316L

## Abstract

The electrosurgical blade is the most common invasive surgical instrument in a cutting and hemostasis process; however, the blade easily leads to the adhesion of overheated soft tissues on the blades and induces a potential danger for the patients. To minimize the adhesive tissues, we proposed the one-step surface texturing method to fabricate anti-adhesive biomimetic scales on stainless steel 316L rapidly based on the self-organized surface microstructures induced by the long-pulse fiber laser, which was inspired by the excellent performances of anti-adhesion and anti-friction in the pangolin scales. The optimal formation parameters, chemical components, and crystal structures of the laser-induced self-organized surface microstructures were investigated in the experiments. Moreover, the underlying formation mechanism was revealed. The electrosurgical blades with biomimetic scales have hydrophobicity and a smaller frictional coefficient, which effectively reduced the adhesion of soft tissue.

## 1. Introduction

Soft tissue adhesion always occurs on the electrosurgical blades during a cutting and hemostasis process in electrosurgery [1]. Although electrosurgical blades have proven effective for minimizing bleeding during surgery, these blades produced high temperature and induced the overheated tissue sticking to the blade, which reduces cutting efficiency and ultimately requires the replacement of the blade; furthermore, it also results in failure of cutting and hemostasis in surgery and induces a potential danger for the patients [2]. Therefore, there is an urgent need to thoroughly resolve the adhesion of the soft tissue on the electrosurgical blades.

Traditional methods to overcome this problem rely on simple coating technology such as diamond-like carbon (DLC) coatings or edge shape optimization technology [3,4,5,6]. Coatings are relatively effective methods to construct a physical barrier between the soft tissue and the instrument surface, which can reduce the soft tissue adhesion on the electrosurgical instrument surface. However, the coatings may decompose particulates and peel off because of high temperature or internal compressive stress [7]. In addition, the coatings are also plagued with fabrication process complexity and increase the cost. Only using edge shape optimization technology is also difficult to resolve the adhesive problem completely [8]. In recent years, surface modification of the electrosurgical blades to reduce tissue adhesion has attracted attention [9,10,11]. Zhang proposed filling the microstructures on the blade surface with biocompatible lubricating liquid silicone oil to reduce adhesion [9]. Lin used a femtosecond laser to produce microstructures on the electrode surface, which can effectively avoid tissue damage [10]. Li used the laser direct writing technology to fabricate the biomimetic microstructures on the blade surface to reduce tissue adhesion and friction [11]. However, these methods involved complex processes or expensive equipment, which limited the applications. 

**Figure 1:**
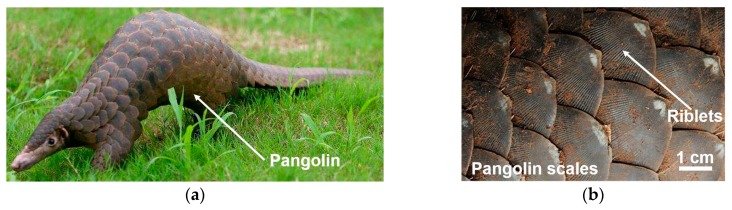
(**a**) Chinese pangolin; (**b**) The pangolin scales with longitudinal ridges on the external surface.

Nature offers a variety of surface structures with remarkable efficiency for tailored functionalities such as anti-adhesion and anti-friction. For instance, the soil-burrowing animal Chinese pangolins, which often dig caves in earth for a living, are covered with scales (Figure 1). The trunk scales on the Chinese pangolin are dark brown with broad rhombic shape; these also have outstanding performances regarding anti-adhesion and anti-friction against soil and rock, except for the protective function [12,13,14]. Both the anti-adhesion and anti-friction are attributed to the corrugated external surface structures of the scales characterized by longitudinal riblets of about 200 µm diameter (Figure 1b) [15,16]. Inspired by the excellent anti-adhesion and anti-friction performances of the pangolin scales, biomimetic surface structures on electrosurgical blade such as pangolin scales are investigated in this work.

Laser-induced self-organized surface structures provide a fast, precise, and low-cost tool for surface microstructuring [17]. The regular self-organized surface structures can have the applications in surface engineering and treatment, notably in tribology, wettability, mechanics, marking, and counterfeiting [18,19,20,21]. A femtosecond laser could induce many types of self-organized micro/nanoscale surface structures on almost all solids [17]. However, a femtosecond laser has the requirement of expensive cost and a temperature-constant clean room, which limits its applications in the industrial field. Given that the long-pulse fiber laser is cheaper and widely used in industry, this work focuses on the self-organized microscale surface structures induced by the long-pulse fiber laser to construct the biomimetic surface structures such as pangolin scales on electrosurgical blades in order to reduce tissue adhesion.

## 2. Materials and Methods 

Stainless steel 316L is widely used in food preparation equipment, marine applications, and medical equipment. 316L is an austenitic stainless steel with a composition given as 62.045%–72% Fe, 16%–18.5% Cr, 10%–14% Ni, 2%–3% Mo, 2% Mn, and 0.03% C and other elements in weight percent. Typically, because of its durability, corrosion, and pitting resistance, surgical tools are usually made of stainless steel 316L [22]. In this work, the experiments were respectively performed on surface-polished commercial stainless steel 316L sheets with thicknesses of 0.64 mm and electrosurgical blades made of stainless steel 316L. Before and after laser radiation, the samples were cleaned ultrasonically with acetone, ethanol, and deionized water successively.

Self-organized surface microstructures on the sample 316L were induced using a fiber laser system (SPI-100C, SPI Lasers Ltd, Southampton, UK) at the wavelength of 1070 nm with the pulse width of 500 μs. The samples were mounted on a computer-controlled XYZ motion platform. During the laser processing, the surface microstructures are fabricated directly by controlling the motion platform with the sample, without the motion of the laser beam. The sample was irradiated by the laser beam normal to the surface, with a focus spot of about 18 μm diameter. The scanning speed of 0.5–5 m/min and a repetition rate of 1000 Hz were used. The fluences in the range of 2000–20,000 J/cm^2^ were applied separately to the 316L sheets and electrosurgical blades. 

The surface morphology of the samples was characterized using a scanning electron microscopy (SEM, FEI Q45, Thermo Fisher Scientific, Waltham, MA, USA) and an optical microscopy. The underlying microchemistry was also studied using energy dispersive spectroscopy during the SEM analyses. The SEM was equipped with an EDS system (EDS, Ametek Inc, Berwyn, PA, USA), and a 25-kV accelerating voltage was applied to the elemental spot analyses of the sample surfaces. Two-dimensional elemental EDS maps were taken of the sample surfaces. In addition, the crystal structures of the sample surfaces were investigated by an X-ray diffractometer (XRD, D-max-2200PC, Japan Rigaku Co., Tokyo, Japan).

The surface wettability of samples was measured using an optical contact angle measuring system (OCA20, DataPhysics Instruments GmbH, Filderstadt, Germany). A 5-μL droplet of deionized water was dispensed onto the sample surface using a syringe, while an image was captured by a camera combined with a 6× magnification system. Then, the contact angle was determined by analyzing the droplet images using the software SCA20. The contact angles on each sample were measured several times, and the average values of the contact angles were obtained.

To study the friction effect on the anti-adhesion performance, the friction coefficients between the blade surfaces and pig’s liver slices were measured using the vertical universal friction and wear testing machine (MMW-1, Ji’nan meta test Ltd., Ji’nan, China). In the machine, the pig’s liver was cut to the disc-shaped slice with the diameter of 45 mm and thickness of 2 mm, which was fixed on a metal substrate. The blade tip was cut into the rectangular bar of 5 mm length, which was fixed on a pin in the machine. In the friction test, the pressure force between the blade surface and liver slice was set to 10 N, and rotation speed of the blade tip on the liver slice was set to 10 r/min according to the cutting parameters in surgery with the lowest measuring error. The friction experiments were carried out at room temperature under ambient conditions. The friction coefficient of each sample was measured at least three times to obtain the average value. 

To evaluate the anti-adhesion effect, a fresh pig’s liver was cut by electrosurgical blades with bionic surface structures. In the test, the cutting speed was 1000 mm/min and the cutting depth was 12 mm. The temperature of electrosurgical knives was kept to 250 °C ± 10 °C during the cutting process. After cutting, the adhesive tissue mass on the electrosurgical blades was weighed. For each electrosurgical blade, the cutting experiments were repeated at least three times, and the average mass of adhesive tissues on the blades were obtained. 

## 3. Results and Discussion

### 3.1. The Self-Organized Surface Structures Similar To Pangolin Scales

**Figure 2:**
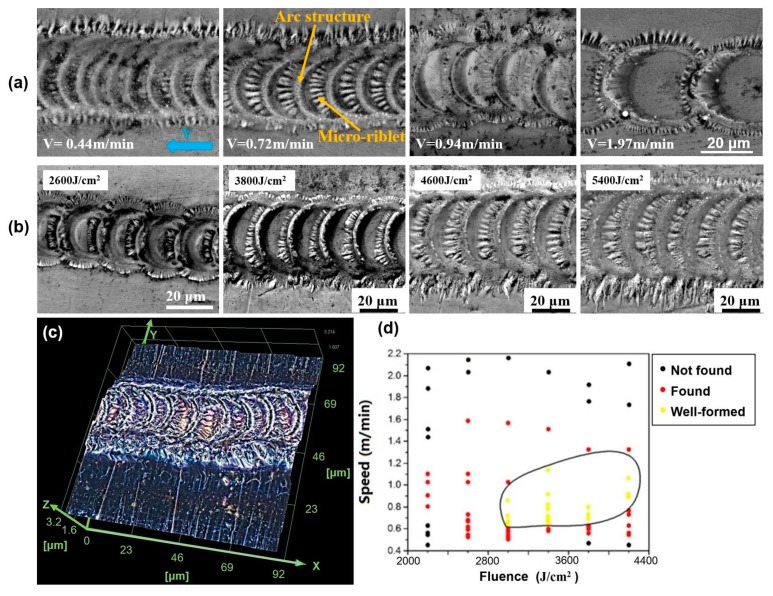
(**a**) SEM images of self-organized surface structures on 316L induced by laser pulses at same fluence of 3400 J/cm^2^ in different moving speeds: 0.44 m/min, 0.72 m/min, 0.94 m/min, and 1.97 m/min respectively (scale bar is 20 μm in all subfigures; blue arrow indicates the direction of the sample movement); (**b**) SEM images of self-organized surface structures induced by laser pulses in the same speed of 1.0 m/min at different the fluence of 2600 J/cm^2^, 3800 J/cm^2^, 4600 J/cm^2^, and 5400 J/cm^2^ respectively (scale bar is 20 μm in all subfigures); (**c**) Three-dimensional morphology of self-organized surface structures in the case of V = 0.72 m/min in Figure 2a; (**d**) The fabrication parameters (fluence, speed) map of the well-formed laser-induced self-organized surface structures on 316L: the data marked in yellow dots indicating the well-formed self-organized surface structures, the data in red dots indicating the poor self-organized surface structures and the data in black dots meaning non-existing self-organized surface structures.

At first, several microgrooves were directly written on the 316L sample surface by laser pulses at different fluences in different moving speeds of samples, as shown in Figure 2a,b. Figure 2a shows the laser-induced surface structures at a fixed fluence Φ = 3400 J/cm^2^ in different moving speeds. In particular, a type of self-organized microstructure was observed in the microgrooves at the speed of 0.72 m/min, which includes arc microstructures filled in the groove and micro-riblets radially distributed along every arc structure, similar to the morphology of pangolin scales. In Figure 2c, a three-dimensional morphology of the self-organized surface structures demonstrates that both the depth of the groove and the height of the arc microstructures are about 0.5 μm, and the height of the micro-riblets is less than 0.5 μm. The self-organized microstructures only formed well in the moderate speeds, as shown in Figure 2a. Furthermore, Figure 2b shows the self-organized surface structures in a fixed speed of 1.0 m/min at different fluence, which implies that micro-riblets only formed well at the moderate fluences. In order to find the fabrication parameters (fluence, speed) zone of the well-formed self-organized surface structures such as the case of 0.72 m/min in Figure 2a, the array of microgrooves were directly written on the 316L sample surface by laser pulses at the fluences of 2000–4400 J/cm^2^ and in the scanning speed of 0.4–2.2 m/min separately. Figure 2d shows the fabrication parameters (fluence, speed) map related to the forming quality of the self-organized surface structures, indicating that the self-organized surface structures formed well in the case of 0.72 m/min in Figure 2a at the fluences of 3000–4200 J/cm^2^ and the speed of 0.6–1.2 m/min.

### 3.2. The Chemical Compositions and Crystal Structures of Self-Organized Surface Structures

**Figure 3:**
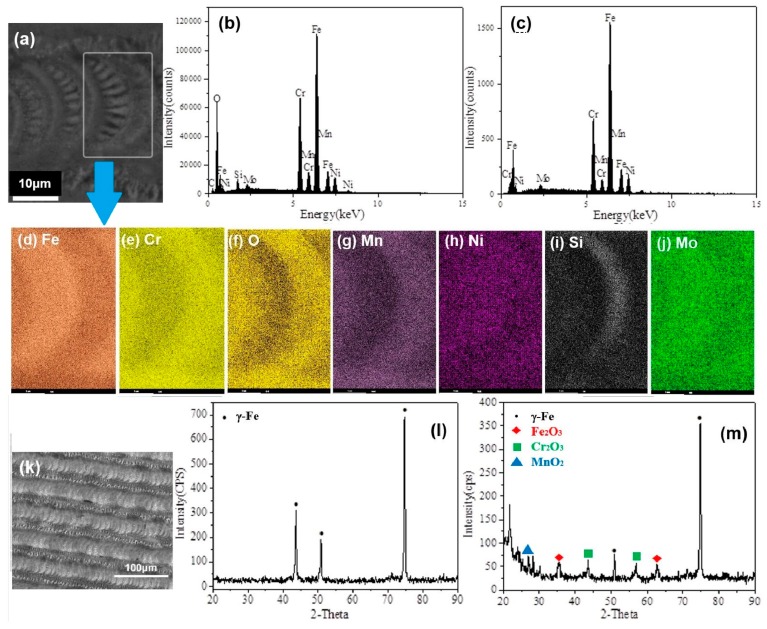
(**a**) SEM image of self-organized surface structures with a white rectangle region for energy dispersive spectroscop (EDS) analysis; (**b**) EDS spectrum of self-organized surface structures; (**c**) EDS spectrum of raw 316L surface without laser irradiation; (**d**–**j**) EDS maps in the white rectangle region in (**a**) showing elemental distributions of iron, chromium, oxygen, manganese, nickel, silicon, and molybdenum respectively; (**k**) SEM image of the large-area self-organized surface structure with 50-µm groove spacing, depicting X-ray diffractometer (XRD) analyzed region; (**l**) XRD spectrum of raw 316L surface without laser irradiation; (**m**) XRD spectrum of self-organized surface structures.

The chemical compositions of the self-organized surface structures were analyzed by energy dispersive spectroscopy (EDS). Figure 3a shows the EDS analyzed region of self-organized surface structures in the case of 0.72 m/min in Figure 2a. Figure 3b,c depict EDS spectra of self-organized surface structures and raw 316L surface without laser irradiation respectively. Comparing two EDS spectra, both the main chemical compositions are nearly same; however, the oxidation is introduced in the self-organized surface structures. Figure 3d–j are two-dimensional elemental EDS maps in the white rectangle region in Figure 3a showing elemental distributions of iron, chromium, oxygen, manganese, nickel, silicon, and molybdenum, respectively. For the micro-riblets of the rectangle region, there are higher contents of iron, nickel, and molybdenum; however, there are lower contents of chromium, oxygen, manganese, and silicon. 

Furthermore, the crystal structures of the self-organized surface structures were investigated by X-ray diffractometer (XRD). Figure 3k shows the XRD analyzed region of the large-area self-organized surface structure with 50 µm groove spacing. Figure 3l,m depict the XRD spectra of the raw 316L surface without laser irradiation and self-organized surface structures, respectively, which implied that the self-organized structures covered with a layer of metallic oxides due to the oxidation, including Fe_2_O_3_, Cr_2_O_3_, and MnO_2_.

### 3.3. The Formation Mechanism of Self-Organized Surface Structures

**Figure 4:**
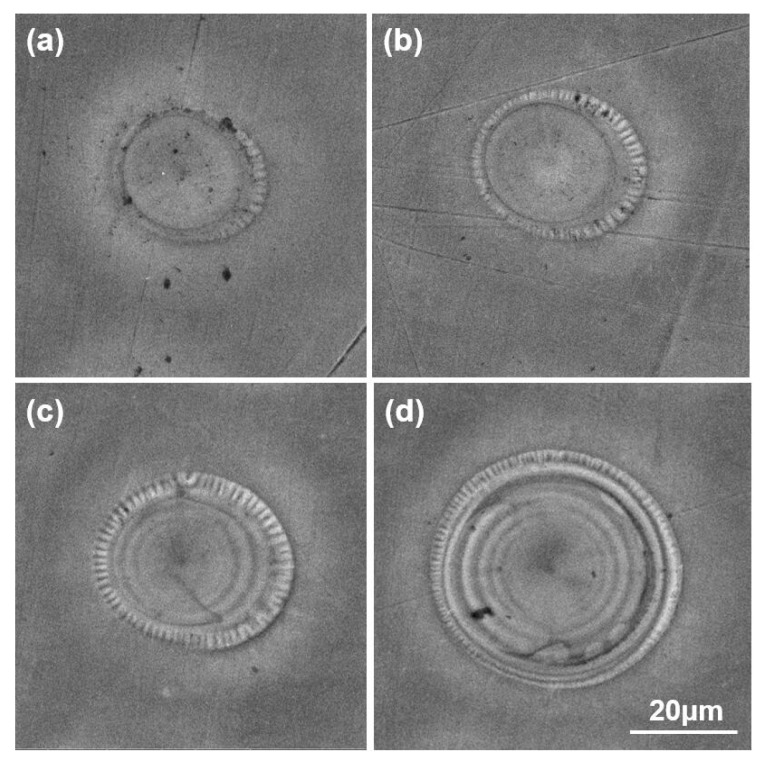
SEM images of surface morphology induced by one laser pulse at the fluence of (**a**) 4200 J/cm^2^, (**b**) 5000 J/cm^2^, (**c**) 5800 J/cm^2^, and (**d**) 6600 J/cm^2^ respectively.

In order to study the formation mechanism of self-organized surface structures induced by long-pulse laser pulses, a stationary 316L sample was shot by one laser pulse. Figure 4 shows the surface morphology induced by one laser pulse at the different fluences of 4200 J/cm^2^, 5000 J/cm^2^, 5800 J/cm^2^, and 6600 J/cm^2^ respectively. In Figure 4d, the depth of crater center is about 0.65 μm and the height of the remelted material is about 2.52 μm, which were measured by a profilometer (Veeco Wyko NT1100, ANFF Co., Kensington, Australia). In Figure 4, after one laser pulse, shallow craters with many short micro-riblets radially distributed along every crater edge appeared on the surface, which belong to laser splashed morphologies [23]. With increasing laser fluence, multiple concentric rings also appeared in the crater in Figure 4c,d. Compared with the self-organized surface structures in Figure 2a,b and considering the low fluence in the well-formed region in Figure 2d, it is reasonable to infer that both the micro-riblets radially distributed along the crater edge in Figure 4, and the micro-riblets radially distributed along every arc-structures in Figure 2a,b are laser splashed morphologies that have the same formation mechanism.

In Figure 2a,b, the self-organized surface structures originated in the laser-driven translative mass redistributions in the overlapping of the irradiated craters. The underlying dynamics involves intricate physical processes including laser-induced absorption, electron–phonon exchange, acoustic relaxation, hydrodynamic motion, quenching, capillary effect, and the recrystallization of molten microfeatures [24]. The formation process is presumed as follows: at the beginning, first a laser pulse produced a molten pool within the focal spot on the sample surface if the laser fluence is larger than the melting threshold. Before the molten pool solidified, the second laser pulse was shot at the overlapping zone of the molten pool, and the molten liquid started to fly outwards under the vapor pressure with the propagation of the expansion waves [21]. In the capillary stage, the surface tension slowed down the moving liquid film and finally stopped its motion to form the micro-riblets radially distributed along the arc microstructure that was the part of the crater edge in the groove. Finally, the molten surface structures recrystallized, and the shape of the splashing morphology was immobilized due to the cooling effect [23,24,25].

For the elemental segregation in the micro-riblets in Figure 3d–j, it can be explained by the rapid cooling effect on the recrystallized microstructures of stainless steel [26]. Since iron, chromium, and nickel are the main elements in weight in 316L, we only discuss the elemental segregation of these three elements here. The micro-riblets originated from splashed morphology, which has higher cooling speed than the substrate material. In the rapid solidification, the transformation from ferrite (δ-Fe) to austenite (γ-Fe) occurs, which results in the elemental segregation [27]. There is an increase in chromium and a corresponding reduction in nickel in the ferrite dendrite core area. Similarly, nickel, an austenite former, concentrated heavily in areas away from ferrite [27]. In addition, lower oxygen in micro-riblets may originate in the splashed liquid moving from the inner part of molten pool that has less oxidation.

### 3.4. Test of Biomimetic Scales on the Electrosurgical Blades

**Figure 5:**
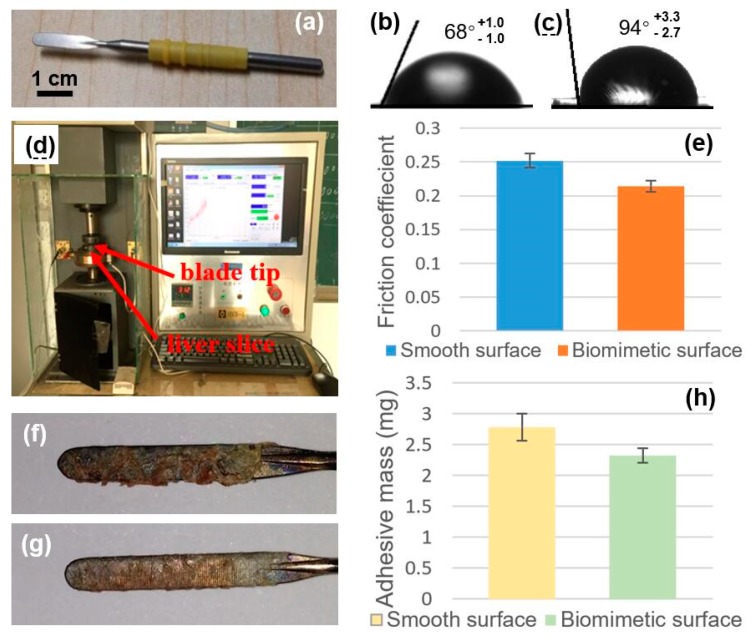
(**a**) Photograph of the electrosurgical knife used in the experiment; (**b**) Optical image of a water drop on the raw 316L plane showing the contact angle; (**c**) Optical image of a water drop on the 316L biomimetic surface showing the contact angle; (**d**) Photograph of the friction testing machine measuring the friction coefficients between the blade surfaces and pig liver slices; (**e**) Friction coefficients between the pig’s liver slices and the blade tips with a smooth surface and biomimetic surface, respectively; (**f**) Optical image of tissue adhesion on the blade with a smooth surface after cutting a pig’s liver; (**g**) Optical image of tissue adhesion on the blade with biomimetic surface after cutting a pig’s liver; (**h**) Tissue adhesion mass on the blades with smooth surface and biomimetic surface after cutting a pig’s liver.

Inspired by the excellent anti-adhesion of the pangolin scales, we fabricated the self-organized surface structures to construct the biomimetic scales on the electrosurgical blades. The electrosurgical blade used in the experiments was shown in Figure 5a. The array of microgrooves with the self-organized surface structures were directly written on the surface of blade tip by laser pulses at the fluences of 3400 J/cm^2^ in the moving speed of 1.2 m/min. On the electrosurgical blade, the array of microgrooves with the spacing of 200 μm is perpendicular to the hilt. Then, the performance of the biomimetic scales on the electrosurgical blades was measured and tested as follows. At first, the surface wettability of samples was studied by measuring the contact angle in ambient conditions. Figure 5b,c show the measured contact angles of the smooth raw 316L sample and biomimetic scales, respectively. The average contact angle of smooth surface was 68°; however, the average contact angle of the biomimetic surface was 94°. Accordingly, biomimetic surface structures can increase the contact angle of 316L samples and transfer the wettability of the 316L samples from hydrophilicity to hydrophobicity. The mechanism of biomimetic scales affecting the contact angle can be explained by the Cassie–Baxter model [28]. The biomimetic scales changed both the roughness ratio of the wet surface area and the fraction of the solid surface area wet by the liquid in the Cassie–Baxter model, resulting in the increased contact angle.

To study the performance of anti-friction, the friction coefficients between the blade tips and pig’s liver slices were measured using the vertical universal friction and wear testing machine (MMW-1), as shown in Figure 5d. Figure 5e shows the measured friction coefficients on the smooth raw 316L sample and biomimetic scales, respectively. Compared with the average friction coefficient 0.2519 on smooth blade surfaces, the biomimetic surfaces have the smaller friction coefficient 0.2143. Therefore, biomimetic surface structures can effectively reduce friction coefficients by 14.9% on average. This is because biomimetic scales have the same anti-friction mechanisms as pangolin scales, including the reduction of effective contact area per unit area, as well as the “guiding effect” and “rolling effect” that were proposed in reference [12,13]. 

Finally, fresh liver was cut by electrosurgical blades with biomimetic scales to evaluate the anti-adhesion effect by weighing the adhesive tissue mass on the blades. After cutting, the adhesive liver tissues on the blades with a smooth surface and a biomimetic surface are respectively shown in Figure 5f,g, which demonstrated that there were significantly more adhesive tissues on the smooth surface than on the biomimetic surface. Figure 5h shows the measured mass of adhesive tissues on the smooth raw 316L sample and biomimetic scales respectively. As a reference, the average mass of adhesive tissues on the smooth blade surfaces was 2.78 mg. However, the average mass of adhesive tissues on the biomimetic surfaces was 2.32 mg. Therefore, we concluded that the biomimetic scales can effectively reduce the adhesion of liver tissue, and the adhesive mass can be reduced by 16.5% on average. The anti-adhesion effect of the biomimetic scales benefits from their hydrophobicity and smaller frictional coefficients.

## 4. Conclusions

In summary, we proposed the one-step surface texturing method to fabricate anti-adhesive biomimetic scales on stainless steel 316L rapidly, based on the self-organized surface microstructures induced by long-pulse fiber laser, inspired by the pangolin scales. The typical self-organized microstructures were observed in the microgrooves written directly by laser pulses, which includes arc microstructures filled in the groove and micro-riblets radially distributed along every arc structure, similar to the morphology of pangolin scales. The self-organized surface structures originated in the laser-driven translative mass redistributions in the overlapping of the irradiated craters. The micro-riblets radially distributed along every arc structure are laser splashed morphology, which formed due to the hydrodynamic motion of the molten metal, quenching, and the capillary effect. Finally, the laser-induced self-organized surface structures were used to construct the biomimetic scales on the electrosurgical blades to minimize the tissue adhesion. After a series of test, biomimetic scales transferred the wettability of the 316L samples from hydrophilicity to hydrophobicity, furthermore effectively reducing the friction coefficients by 14.9% on average. After evaluation, biomimetic scales can significantly reduce the adhesion of soft tissue by 16.5% on average. This work may provide a new insight into the fast, low-cost fabrication of anti-adhesive microstructures on stainless steel 316L in the electrosurgical instruments.
